# Glycated and Non-Glycated Human Alpha-1 Antitrypsin in Hyperglycemic Wound Healing: In Vivo and In Vitro Models

**DOI:** 10.3390/biology15080606

**Published:** 2026-04-11

**Authors:** Idan Farber, Alon Naumchik, Yosef Istoyler, Melody Zaknoun, Yuval Anav, Lihie Sheffer, Ronen Schuster, Dor Halpern, Vladimir Fridman, Merav Cohen-Lahav, Samuel Cohen, Eli C. Lewis, Eldad Silberstein

**Affiliations:** 1 Department of Clinical Biochemistry and Pharmacology, Faculty of Health Sciences, Ben-Gurion University of the Negev, Be’er Sheva 84101, Israel; alonnaum@post.bgu.ac.il (A.N.); istoyler@post.bgu.ac.il (Y.I.); yuvaltal.8@gmail.com (Y.A.); lihieshe@post.bgu.ac.il (L.S.); ronen.schus@gmail.com (R.S.); halpern.dor1@gmail.com (D.H.); fridmanv@gmail.com (V.F.); cohensam@post.bgu.ac.il (S.C.); eldads@bgu.ac.il (E.S.); 2 Plastic and Reconstructive Surgery Department, Soroka University Medical Center, Be’er Sheva 84101, Israel; 3 Laboratory of Management, Soroka University Medical Center, Be’er Sheva 84101, Israel; meravcl@clalit.org.il

**Keywords:** diabetes, wound healing, hyperglycemia, alpha-1 antitrypsin, glycation, inflammation, diabetic wounds, tissue repair, macrophages, re-epithelialization

## Abstract

Impaired wound healing is a major cause of morbidity among patients with diabetes. Human α1-antitrypsin (hAAT) promotes the resolution of injured tissues. In hyperglycemic conditions, circulating hAAT is likely to undergo glycation, yet it is unknown whether its reparative properties are preserved. We hypothesized that clinical-grade hAAT treatment, but not deliberately glycated hAAT (gly-hAAT), would promote wound repair under hyperglycemic conditions. Mice were rendered hyperglycemic, excisional wounding was performed, and wounds were treated with topical albumin or hAAT every three days. The wound area was assessed, and samples were collected for histology and gene expression analysis. Gly-hAAT was generated from clinical-grade hAAT, after which in vitro RAW 264.7 macrophage responses and re-epithelialization of A549 cells were assessed. Gap closure was further assessed using sera from a human cohort (prospective samples from 10 patients with poorly controlled diabetes at Soroka University Medical Center, Beer-Sheva, Israel, 2018). Group comparisons were performed using one-way ANOVA with Tukey’s post hoc test. hAAT accelerated in vivo wound closure and in vitro A549 cell gap closure, accompanied by an anti-inflammatory IL-1Ra/IL-1β gene expression profile. In contrast, gly-hAAT inhibited normoglycemic mouse wound closure, evoked an inflammatory response in macrophages, and interfered with A549 cell gap closure; concomitant hAAT treatment improved gap closure. Similarly, patient serum inhibited A549 gap closure, and concomitant hAAT treatment improved gap closure. Importantly, inferential statistical analysis was not performed on this outcome due to the small and heterogeneous human cohort. In conclusion, hAAT accelerated wound closure in hyperglycemic mice and in A549 cells, whereas gly-hAAT promoted inflammatory responses and impaired wound closure, a trend reversed by native hAAT. These findings support the concept that glycation undermines the beneficial functions of circulating hAAT and provides a mechanistic insight into the pathophysiology of diabetic wound healing. Further studies are warranted to evaluate clinical-grade hAAT as a potential therapeutic for hyperglycemia-associated impaired wound healing.

## Simple Summary

Chronic wounds are a major complication of diabetes and may lead to infection, hospitalization, and limb amputation. High blood sugar creates a harmful environment that delays tissue repair. In this study, we examined whether human alpha-1 antitrypsin (hAAT), a natural blood protein with anti-inflammatory and tissue-protective effects, could improve wound healing under high-glucose conditions. We also tested whether exposure to glucose alters its function and reduces its beneficial activity. Using mouse wound models, cell culture experiments, and serum samples from patients with poorly controlled diabetes, we found that treatment with non-modified hAAT improved wound closure and promoted more effective tissue repair. In contrast, glucose-modified hAAT triggered inflammatory responses and impaired healing. In some experiments, adding the non-modified hAAT reduced the harmful effects of glucose-modified hAAT or diabetic serum. These findings suggest that high glucose may impair the protective role of hAAT in wound healing, while treatment with clinical-grade hAAT may help restore a more favorable healing environment. These findings support the development of new treatments for diabetic wounds.

## 1. Introduction

The skin’s main function is to shield the body from environmental insults and prevent fluid loss. Therefore, upon injury, it is extremely important to restore skin integrity in a rapid and efficient manner. The physiological wound healing process is initiated by an inflammatory surge characterized by inflammatory cytokine production, including IL-1β, TNFα, and IL-6, as well as the desirable migration of neutrophils and facilitation of inflammatory M1 macrophages for the elimination of pathogens and removal of cell debris [1]. While the inflammatory wave activates fibroblasts, drives re-epithelialization, and promotes angiogenesis and revascularization, it must subside in order to allow these processes to unfold. Difficult-to-heal wounds occur when inflammatory triggers persist, be it infection, residual inflammatory agents, ischemia, or mechanical disruption. Hyperglycemia represents a persistent inflammatory trigger and often causes difficult-to-heal wounds [2]; as such, it poses a significant risk for infections and lower limb amputations. Wounds that develop in individuals with poor glycemic control depict inadequate revascularization, dysfunctional fibroblasts, endothelial and epithelial cells [2], a predominance of M1 macrophages that typically express inducible nitric oxide synthase (iNOS), and, at times, a bacterial biofilm [3].

The molecular and cellular profile of a desired inflammatory resolution process includes M2-like macrophages, which typically express arginase-1 (Arg-1) and CD206, and anti-inflammatory cytokines, such as IL-1 receptor antagonist (IL-1Ra) [4]. The latter mitigates IL-1–elicited inflammatory processes by preventing inflammatory IL-1 family members from activating the IL-1 receptor [5]. Thus, the IL-1Ra/IL-1β ratio is of significance to wound repair outcomes [6].

Human α1-antitrypsin (hAAT) is a 52 kDa circulating glycoprotein that belongs to the serine protease inhibitor family [7]. Its circulating levels rely on liver production, while mucosal tissues produce local hAAT. From steady-state serum levels, hAAT is elevated by infection, inflammation, hypoxia, and tissue injury, remaining high for a week or more. It is also elevated during a healthy pregnancy. hAAT promotes multiple processes relevant to wound healing. hAAT promotes mature blood vessel development [8], decreases the amount of necrotic tissue [9], and accelerates re-epithelialization both in vitro and in vivo [10,11]. Interestingly, corticosteroids limit the beneficial effects of hAAT, supporting its classification as an inflammation-driven pro-resolution agent [10,11].

hAAT inhibits the activity of inflammatory serine proteases, such as neutrophil elastase, which would otherwise degrade tissue elastin, activate stimulatory protease-activated receptors (PARs) on immunocytes, and activate pro–IL-1β released from injured cells [7,12]. Accordingly, the major clinical characteristic of genetic hAAT deficiency (AATD) is lung alveolar wall deterioration [13]. Although less common, patients with AATD exhibit dermatological manifestations, such as panniculitis, vasculitis, psoriasis, urticaria, and angioedema; they also suffer from impaired wound healing and longer hospitalization times [14]. Despite reducing inflammation, hAAT is an immunomodulator that directs, rather than blocks, neutrophils, macrophages, dendritic cells, B lymphocytes, and, albeit indirectly, T and NK cells toward tissue-protective phenotypes [15,16].

In addition to inhibiting particular proteases, hAAT acts by binding local agents [17]. For example, hAAT increases the production of inflammation-induced IL-1Ra in a manner independent of protease inhibition [12] and binds to the neutrophil chemokine, IL-8, and to several danger-associated molecular pattern molecules (DAMPs) [15,17]. Additionally, a cysteine residue on its surface covalently binds to local nitric oxide and turns hAAT anti-bacterial [18]. In this regard, the integrity of its surface amino acids is cardinal for its function.

The non-enzymatic time-dependent covalent attachment of glucose to lysine and arginine residues of a protein yields advanced glycation end-products (AGEs) [19]. AGEs are inflammatory; they induce IL-1β, TNFα, and IL-6 production, sustain inflammatory signaling [20,21,22], and impair wound healing [23].

Studies of diabetic wound exudates have shown that local AAT is degraded and non-functional [24] and that AAT levels are lower compared to acute normoglycemic wounds [25]. Because AAT is synthesized in the liver and circulates in the bloodstream, it is exposed to glucose both during production and throughout its plasma lifetime, making it susceptible to glycation. Like other abundant circulating proteins, AAT contains surface-exposed lysine and arginine residues that serve as targets for non-enzymatic glycation. Similar glycation processes occur in other proteins, most notably hemoglobin (HbA1c) and albumin (glycated albumin). Beyond serving as clinical markers, glycation alters protein structure and function. Indeed, glycated albumin acquires pro-inflammatory properties [26,27].

Given these considerations, it remains unclear whether glycation alters AAT’s broader functions, including its role in tissue repair. The present study, therefore, examines whether glycation acts as a functional switch, whereby hAAT shifts from a pro-resolution, tissue-protective mediator to a pro-inflammatory molecule. It also evaluates whether naïve hAAT supplementation supports wound healing in a hyperglycemic environment.

## 2. Materials and Methods

### 2.1. Animals

All animal experiments were conducted at Ben-Gurion University of the Negev (Beer-Sheva, Israel, 2024). Studies were approved by the Institutional Animal Care and Use Committee (BGU302-1-2024D, 14 January 2024) and conducted in line with the Guide for the Care and Use of Laboratory Animals, 8th Edition. C57BL/6 mice (8–12-week-old females, 18–22 g, Envigo Laboratories, Inc., Rehovot, Israel) were housed at a standard vivarium. Mice transgenic for human AAT (hAAT^+/+^; C57BL/6 background, females, 18–22 g) were generated at the University of British Columbia, Canada, and bred in-house [28]. hAAT^+/+^ mice express constitutive levels of circulating hAAT (<1 µg/mL) and are used as a positive control for hAAT treatment. Female mice were used to maintain sex uniformity across the experimental groups.

### 2.2. In Vivo Wounds in Hyperglycemic Mice

Mice were weighed before interventions and periodically thereafter. Hyperglycemia was induced using streptozotocin [29,30] (STZ, Sigma-Aldrich, Rehovot, Israel) in 50 mM sodium citrate, pH 4.5. For the transgenic experiment, WT and hAAT^+/+^ mice were rendered hyperglycemic using STZ (225 mg/kg; *n* = 4 per group), alongside normoglycemic WT controls that did not receive STZ (*n* = 4). This higher-dose regimen was initially used for proof-of-concept purposes to ensure robust hyperglycemia induction. However, it resulted in impaired wound closure in hyperglycemic WT mice, necessitating early euthanasia according to ethical guidelines. Consequently, subsequent topical treatment experiments were performed using a lower STZ dose (100 mg/kg). This regimen induced a more moderate hyperglycemic phenotype associated with delayed wound healing while preserving animal viability and allowing longitudinal follow-up. Animals in this experiment were allocated to the following groups: normoglycemic untreated controls (CT, *n* = 22), hyperglycemic albumin-treated mice (ALB, *n* = 15), and hyperglycemic hAAT-treated mice (hAAT, *n* = 13); data were pooled from 4 independent trials. Mice received a single intraperitoneal injection of STZ at a volume of 250 µL per mouse. Blood glucose levels were measured daily from tail-tip blood using a standard handheld glucometer. Measurements were obtained under two conditions: after a 12 h fast (following food withdrawal from the cages) or as random non-fasting measurements during the day. Hyperglycemia was defined as ≥120 mg/dL fasting or ≥250 mg/dL non-fasting blood glucose. One week into hyperglycemia, mice were anesthetized, and the shaved dorsal skin was disinfected. A surgical excision was created at the center of the dorsum using a sterile biopsy punch. In the initial proof-of-concept experiment using transgenic mice, a 5 mm punch was used. In the subsequent topical treatment experiments, an 8 mm punch was used, as the larger wound size enabled clearer visualization and more reliable quantification of treatment-related differences in wound closure. To account for minor unavoidable inter-animal differences in baseline wound geometry, wound closure was expressed as a percentage of the initial wound area. Human serum albumin (70024-90-7, Sigma-Aldrich, Rehovot, Israel) or hAAT (Glassia^®^, Kamada Ltd., Ness Ziona, Israel) was introduced directly to the wound borders (4 mg/kg in 100 µL) [7] on the day of excision and then every 3 days. Wounds were photographed serially, and images were analyzed using ImageJ (MedCalc Software, Version 1.53e, Ostend, Belgium). Animals were followed until complete wound closure, which generally occurred between days 10 and 12 post-wounding.

Histological and gene-expression analyses were performed for the topical hAAT treatment experiment with tissue harvested on day 3 post-wounding. These analyses were performed in separate experiments to avoid interfering with serial wound closure assessment. The experimental groups included WT mice (*n* = 8), hAAT-treated mice (*n* = 8), and ALB-treated mice (*n* = 5). Data was pooled from two independent trials.

Animals were included in the study upon successful induction of hyperglycemia. Exclusion criteria were established a priori according to the approved animal care protocol and included clinical signs of severe distress, dehydration, or diabetic ketoacidosis, such as lethargy, ruffled fur, hunched posture, reduced mobility, or significant weight loss, as well as lack of wound healing progression for more than one week.

### 2.3. In Vivo Wounds in Normoglycemic Mice Treated with Glycated hAAT

Normoglycemic WT C57BL/6 mice underwent surgical excisional wounding at the center of the shaved dorsum as described above. Mice were allocated to three treatment groups (*n* = 5 per group). Human serum albumin, glycated hAAT (gly-hAAT), or a combination of gly-hAAT supplemented with naïve hAAT was introduced directly to wound borders (4 mg/kg in 100 µL) on the day of excision and every three days thereafter. Wounds were photographed serially, and images were analyzed using ImageJ (MedCalc Software, Version 1.53e, Ostend, Belgium).

### 2.4. Anesthesia, Analgesia, and Euthanasia

Mice were anesthetized with inhaled isoflurane. Induction was performed in an induction chamber (2.5% isoflurane), followed by maintenance via an inhalation mask (2.0% isoflurane). Depth of anesthesia was monitored by respiratory rate. Post-operative analgesia was provided with dipyrone (metamizole; Optalgin) at 200 mg/kg (administered via drinking water; 1 mL per 250 mL H_2_O; replaced every 3 days). For tissue collection, mice were euthanized in a CO_2_ chamber. Death was confirmed by the absence of spontaneous respiration and heartbeat and a lack of response to a firm toe pinch, in accordance with accepted veterinary guidelines.

### 2.5. Randomization and Blinding

Animals were randomly selected from multiple cages of the same strain, sex, and age range and arbitrarily assigned to experimental groups prior to interventions. Following grouping, mice were returned to identical housing conditions and received uniform supportive care. Routine animal handling, including bedding replacement, food and water supply, and post-operative analgesia, was performed by staff members who were blinded to group allocation. Wound image analysis and outcome quantification were performed by an independent investigator blinded to treatment assignment. All treatments were administered in identical volumes using insulin syringes (300 µL) and delivered to the same wound locations to minimize procedural variability.

### 2.6. Histological Analysis

Animals were sacrificed; wounds were excised and immediately immersed in 10% neutral-buffered formalin (Sigma-Aldrich). Tissues were sectioned at 4–6 μm, mounted on slides, and stained with Hematoxylin and Eosin (H&E; Jackson ImmunoResearch, West Grove, PA, USA).

### 2.7. Glycation of hAAT in Acellular Conditions

Non-enzymatic glycation of hAAT was conducted, adapting the methodology described by Duell et al. [31]. Briefly, 0.5 mL of hAAT (0.59 mg protein) in 2 mL of 50 mM Tris-HCl buffer (pH 8.0) was combined with 12 mg/mL of sodium borohydride. D-glucose was then added to concentrations of 400, 500, 800, and 4000 mM; the preparations were sealed under nitrogen and incubated at 37 °C for 7 days. Following incubation, glycated preparations underwent extensive dialysis against the original buffer, followed by centrifugal size-exclusion filtration to remove residual glucose. The degree of protein glycation was evaluated using a fructosamine assay, which detects ketoamine adducts formed by non-enzymatic glycation of protein amino groups [32,33]. The assay was performed in the biochemistry laboratories of Soroka University Medical Center using a standard clinical protocol.

To optimize the in vitro glycation model, hAAT preparations generated under the different glucose concentrations were compared with glycated albumin analyzed under the same fructosamine-based analytical framework. Based on these analyses, the 800 mM glucose condition was selected for subsequent experiments. This condition generated a clearly detectable and reproducible glycation signal in hAAT within the range observed for glycated albumin, while leaving relatively low residual free glucose after purification.

### 2.8. Western Blot Analysis

Sample protein concentrations were determined using the BCA protein assay kit (Cat#202389, Santa Cruz Biotechnology). For each sample, 20 µg of total protein was loaded and resolved on Tris-glycine SDS-PAGE using a 10% resolving gel with a stacking gel. Proteins were electrotransferred onto nitrocellulose membranes (Cat#1620147, Bio-Rad, Hercules, CA, USA). Membranes were blocked with 5% BSA in Tris-buffered saline containing 0.1% Tween 20 (TBS-T) for 45 min at room temperature and then incubated with mouse anti-human AAT (1:1000, Cat#VMA00662, Bio-Rad), followed by goat anti-mouse HRP-conjugated secondary antibody (1:10,000, Cat#STAR207P). Bands were visualized using enhanced chemiluminescence (ECL, Cat#XLS063, Cyanagen, Bologna, Italy). Western blot analysis was performed in two independent experiments. Coomassie Blue staining was used to document overall protein loading, and full, uncropped blots are provided in Supplementary Figure S1.

### 2.9. Elastase Activity Assay

Neutrophil elastase activity was determined in acellular conditions using a designated kit (Sigma-Aldrich, St. Louis, MO, USA), according to the manufacturer’s instructions. Briefly, elastase (0.39 μM) was incubated with hAAT and gly-hAAT at the indicated concentrations, and the kinetics of the cleaved color product were determined.

### 2.10. In Vitro Epithelial Gap Repair Assay (Scratch Assay) and Macrophage Stimulation Assay

The in vitro scratch assay was performed using A549 cells (Cat# CCL-185, ATCC) grown to confluence in 24-well plates and then scratched uniformly using a 200 µL pipette tip, creating a cell-free area [10]. Cultures were then washed twice with complete RPMI 1640 supplemented with 2.5% FCS (both from Biological Industries Inc., Beit Haemek, Israel), and serial images were acquired by a photomicroscope (Zeiss, Oberkochen, Germany). Cell-free areas were analyzed by ImageJ.

RAW 264.7 cells (ATCC, Cat#SC-6003) were seeded in 48-well plates (1 × 10^5^ cells/well) in RPMI 1640 medium supplemented with 5% FCS. Cells were incubated for 4 h with indicated concentrations of hAAT or gly-hAAT and then stimulated with 5 ng/mL LPS. Eight-hour TNFα levels were determined by DuoSet ELISA (Cat#DY410, R&D systems, Minneapolis, MN, USA). All measurements were performed in triplicate.

### 2.11. Gene Expression Analysis

Wound samples were submerged in RNA Save (Biological Industries), homogenized in a polytron homogenizer, and loaded onto RNase-free microcentrifuge tubes. RNA was extracted using Gynzol^®^ reagent (Invitrogen, Waltham, MA, USA) and isolated on columns (RNAqueous^®^-Micro, Thermo Fisher Scientific, Waltham, MA, USA), following the manufacturer’s guidelines. Eluted RNA was quantified using NanoDrop (Wilmington, DE, USA), and 200 ng were reverse-transcribed using the Prime Script RT Reagent kit (Quanta Biotech, San Francisco, CA, USA). Quantitative PCR was performed at a 20-μL volume reaction using an RT-PCR system (StepOnePlus™ Real-Time PCR, ThermoFisher Scientific Corporation, Waltham, MA, USA) and SYBR Premix Ex Taq II (Quanta Biotech, San Francisco, CA, USA). CFX96 manager software, Version 3.1, was used to determine threshold cycle values; β-actin was used as a reference gene. Primers were designed for murine transcripts as follows: IL-1β ‘5-CTTCCAGGATGAGGACATGAAGG-3′ (forward), ‘5-AGTGCAGTTGTCTAATGGGA-3′ (reverse); VEGF, 5′-TGGGACTGGATTCGCCATTT-3′ (forward), 5′-GTGGGTGGGTGTGTCTACAG-3′ (reverse); IL-1Ra, 5′-GACCCTGCAAGATGCAAGCC-3′ (forward), 5′-GAGCGGATGAAGGTAAAGCG-3′ (reverse); MCP-1 5′-AGGCATCACAGTCCGAGTCA-3′ (forward), ‘5-CCACAACCACCTCAAGCACT-3′ (reverse), ARG-1 5′-AACACGGCAGTGGCTTTAACC-3′ (forward), ‘5-GGTTTTCATGTGGCGCATTC′ (reverse), CD206 5′-GGCTGATTACGAGCAGTGGA-3′ (forward), ‘5-CATCACTCCAGGTGAACCCC-3′ (reverse), CD14 5′-CAGAGAACACCACCGCTGTA-3′ (forward), ‘5-ACACGCTCCATGGTCGGT A-3′ (reverse) and β-actin 5′-CATTGCTGACAGGATGCAGA-3′ (forward), ‘5-TGCTGGAAGGTGGACAGTGA-3′ (reverse).

### 2.12. Human Serum Collection and Analysis

Human serum samples were used to provide an initial exploration of their impact, as well as the effects of concomitantly added clinical-grade hAAT, on in vitro assays. Serum samples were prospectively collected at Soroka University Medical Center (Beer-Sheva, Israel, 2018) during routine clinical diabetes care for predefined experimental analyses in the present study. In vitro experiments were performed at the Department of Clinical Biochemistry and Pharmacology, Faculty of Health Sciences, Ben-Gurion University of the Negev.

The study was approved by the Institutional Review Board of Soroka University Medical Center, Israel (SOR 1106-2018, 20 January 2018), in accordance with ethical guidelines for biomedical research.

After informed consent was obtained, 10 adult patients (mean age 60 ± 7.2 years) diagnosed with poorly controlled diabetes (HbA1c ≥ 7.5%) and eligible for limb amputation were recruited for serum sampling at the diabetes clinic. Exclusion criteria included acute systemic infection, immunosuppressive therapy, or refusal to provide informed consent. Glycated hemoglobin (HbA1c) and fasting blood glucose levels were measured. In addition, serum samples were obtained from 3 healthy adult donors and used as reference controls in the serum gap-closure assays.

Sera were added to the epithelial scratch assay at a 1:10 dilution. The assay was performed in the presence of 5% FCS, as reduced closure rates were anticipated. The gap area was quantified at the indicated time points and expressed as a gap area (% of the initial area at time 0). The area under the curve (AUC) was calculated over 0–24 h using the trapezoidal rule in GraphPad Prism. ΔAUC was defined as the difference between the AUC of the experimental condition and the corresponding reference condition.

### 2.13. Statistical Analysis

Statistical significance between groups was evaluated using one-way ANOVA followed by Tukey’s post hoc test for multiple comparisons. Before ANOVA, the assumptions of independence of observations, approximate normal distribution of the data/residuals, and homogeneity of variances across groups were assessed. For the human serum experiments, gap closure over time was summarized by calculating the area under the curve (AUC, 0–24 h) using the trapezoidal rule. Because of the small sample size and clinical heterogeneity of the human cohort, these analyses were considered exploratory and primarily descriptive. Accordingly, the results were interpreted cautiously and were not used as the basis for definitive inferential conclusions. A *p*-value < 0.05 was considered statistically significant. Statistical processing was performed using GraphPad Prism software (GraphPad Software, Version 9, La Jolla, CA, USA).

## 3. Results

### 3.1. Circulating Transgenic hAAT Accelerates Wound Closure in Hyperglycemic Mice

**Figure 1:**
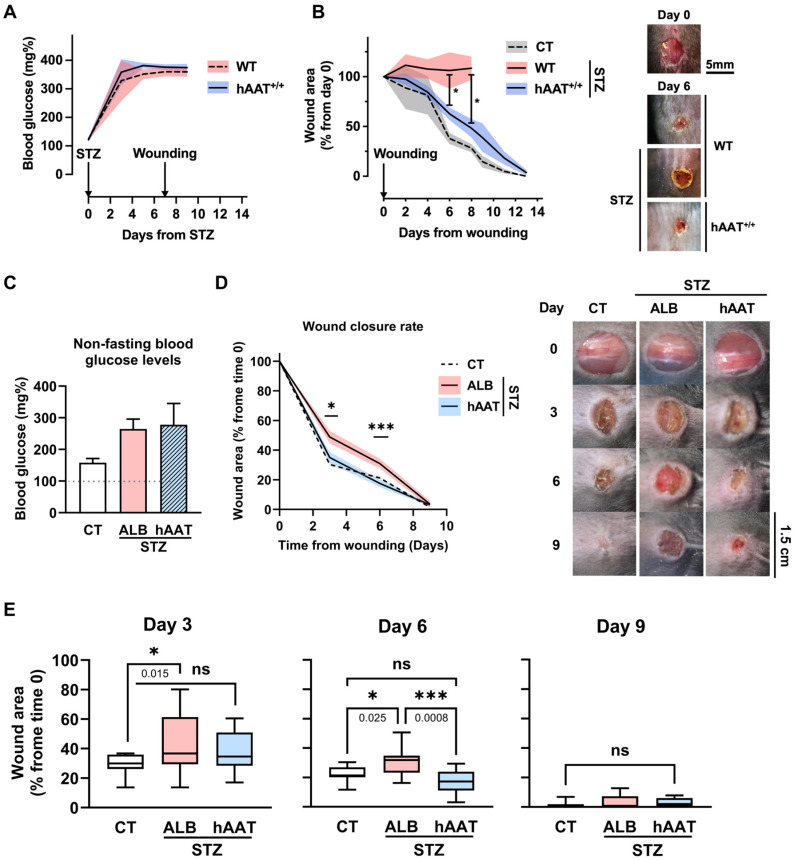

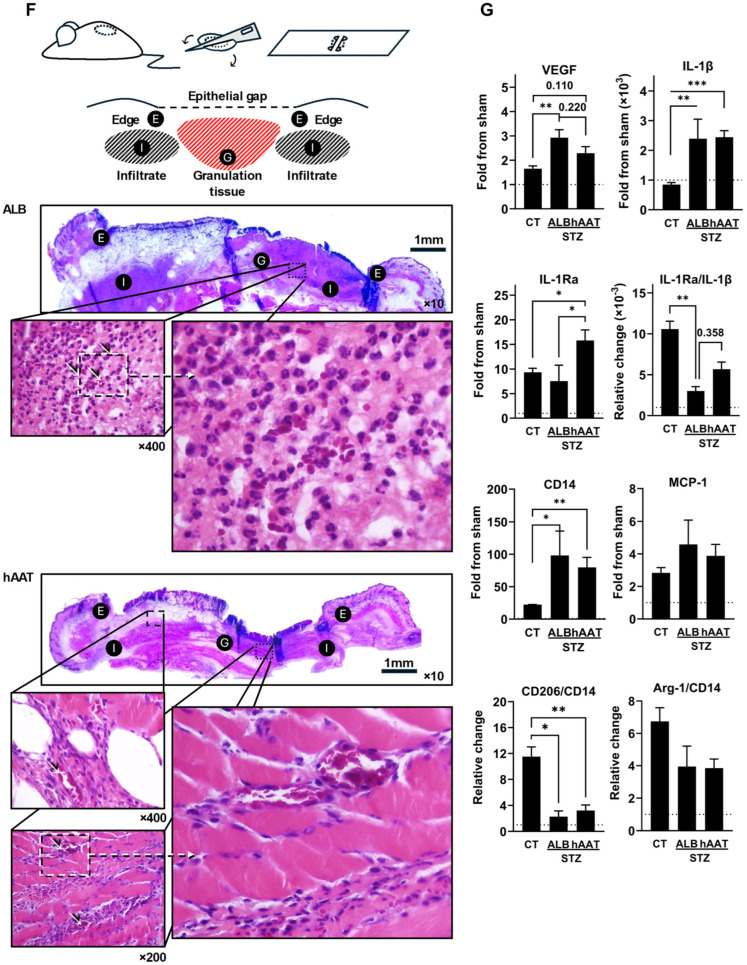
Effect of hAAT on wound healing in hyperglycemic mice: (**A**,**B**) Circulating transgenic hAAT. (**A**) Hyperglycemia evoked by STZ (225 mg/kg) in WT and hAAT^+/+^ mice (*n* = 4 mice per group), alongside normoglycemic CT mice not treated with STZ (*n* = 4 mice per group). Data was generated in two independent experiments. (**B**) Non-fasting blood glucose levels. Mean ± SEM; deviation values marked by solid fill; * *p* < 0.05. (**Right**), representative images. (**C**–**G**) Topical clinical-grade hAAT. Treatment groups were induced to develop hyperglycemia using STZ 100 mg/kg, and animals were wounded a week into hyperglycemia. Animals underwent excision wounds using a punch biopsy (8 mm). The control group was wounded without induction of hyperglycemia (CT, *n* = 22 biological replicates). Treatments included intradermal infiltration of 2 mg/100 µL albumin (ALB, *n* = 15 mice per group) or hAAT 2 mg/100 µL (hAAT, *n* = 13 mice per group) at the time of wounding and every 3 days. Data pooled from 4 independent trials. For histological and gene expression analyses, separate experiments were conducted, with wound tissues harvested on day 3 post-wounding (WT, *n* = 8; hAAT, *n* = 8; and ALB, *n* = 5 mice per group). (**C**) Blood glucose levels on the day of wounding. Mean ± SEM (**D**) Wound closure follow-up. Data presented as % from the initial wound area. mean ± SEM, deviation values marked by solid fill. (**Right**)—representative images on the indicated days, * *p* < 0.05, *** *p* < 0.001. (**E**) Wound closure grouped by day. Box plots, mean and whiskers (min to max); * *p* < 0.05, *** *p* < 0.001 between groups per time point. *ns, not significant*. (**F**) Day 3 histology. Illustration of tissue elements related to wound repair. (**Below**) are representative microscopic images of H&E-stained wound site samples collected from hyperglycemic mice on day 3 from wounding. Albumin-treated hyperglycemic wounds show dense inflammatory infiltrates and extravasated erythrocytes within the granulation tissue, without clear evidence of mature blood vessel formation. In contrast, hAAT-treated wounds display more organized tissue architecture, more orderly inflammatory cell distribution, and visible mature blood vessels containing erythrocytes. *ALB arrows*—extravasated erythrocytes; *hAAT arrows*—erythrocytes contained within blood vessels. Magnification panels are shown at ×10 digital amplification with a 1 mm scale bar. (**G**) Day 3 relative gene expression. Data presented as fold from sham-operated mice (dashed line). CT, normoglycemic mice. Mean ± SEM; * *p* < 0.05, ** *p* < 0.01, *** *p* < 0.001.

The impact of hAAT therapy on wound closure under hyperglycemic conditions was addressed using WT and hAAT^+/+^ mice. As shown (Figure 1A), hyperglycemia was comparable between groups at the time of implementing a 5 mm dorsal excisional wound (Figure 1B). Normoglycemic WT mice (CT) began closing wounds after day 4 from wounding (wound area 81.50 ± 38.91 percent from day 0 compared to 38.00 ± 11.14% on day 6, mean ± SEM, *p* < 0.05); hyperglycemic WT mice did not close wounds for at least 8 days, after which follow-up was discontinued for ethical concerns. Hyperglycemic hAAT^+/+^ mice initiated wound closure between days 2 and 4, albeit without reaching statistical significance compared to hyperglycemic WT mice at those time points. Nonetheless, the two hyperglycemic groups diverged on days 6 and 8, during which hAAT^+/+^ mice exhibited a clear capacity for wound closure (WT and hAAT^+/+^ wound area day 6, 106.30 ± 36.57% and 62.75 ± 12.50%, and day 8, 108.5 ± 23.63% and 48.25 ± 18.52%, mean ± SEM, *p* < 0.05).

### 3.2. Topical Clinical-Grade hAAT Accelerates Wound Closure in Hyperglycemic Mice

After hAAT^+/+^ mice provided a proof-of-concept for improved wound closure under hyperglycemic conditions, the possibility of local hAAT treatment was tested herein with WT mice. Human albumin was used at the same dose and timing to control for treatment. Low-dose STZ was applied, and an 8 mm excisional wound was performed so as to reduce morbidity and allow a longer follow-up. As shown in Figure 1C, hyperglycemia was comparable between groups. According to imaging follow-up (Figure 1D), hyperglycemic albumin-treated mice exhibited poor wound closure (normoglycemic and albumin-treated hyperglycemic mice day 3 wound area 30.35 ± 6.36% and 43.31 ± 19.35% from initial wound, day 6 wound area 22.80 ± 5.22% and 30.97 ± 9.86%, *p* = 0.015 and *p* = 0.022 per time point, respectively). In contrast, topical hAAT resulted in a significantly more robust healing response (day 6, 17.55 ± 7.98%, *p* < 0.001 from day 6 for the albumin group). A day-by-day comparison is provided in Figure 1E.

According to histological analysis of day 3 wound samples (Figure 1F), the hyperglycemic control group exhibited inflammatory infiltrates. Within the granulation tissue, extravasated red blood cells were observed in the absence of concomitant mature blood vessel formation. In contrast, wounds treated with hAAT exhibited a structured organization of the tissue, orderly regions of inflammatory infiltration, and mature blood vessels carrying red blood cells, which are clearly evident.

Day 3 wound samples were also collected for gene transcript analysis. This time point was selected to capture molecular events preceding the phenotypic divergence observed between treatment groups, particularly differences in wound closure that became evident by day 6.

As shown in Figure 1G, compared to samples from normoglycemic mice, wounds from hyperglycemic mice treated with albumin exhibited significantly elevated transcript levels of VEGF and IL-1β. Mice treated with hAAT displayed VEGF transcript levels that were comparable to normoglycemic mice, yet IL-1β transcript levels were comparable to albumin-treated hyperglycemic wounds. Unlike VEGF and IL-1β transcript levels, IL-1Ra transcript levels were significantly higher in hAAT-treated wounds, and the IL-1Ra/IL-1β ratio per animal was predominantly IL-1Ra. IL-1Ra/IL-1β is used to infer the status of resolution pathway trends without protein-level validation, thus maximizing data extraction from minuscule samples. According to analysis of macrophage-related genes, wound samples from hyperglycemic mice exhibited greater CD14 transcript levels, representing macrophagic burden, as well as a greater level of MCP-1 transcripts, irrespective of wound treatment. Transcript levels of CD206 and Arg-1, normalized to CD14 transcripts, provide insight into the presence of M2-like macrophages. As shown, hyperglycemic wounds lack these markers, and treatment with hAAT did not alter this profile at this early time point.

### 3.3. Glycated hAAT (gly-hAAT) Is Inflammatory

**Figure 2:**
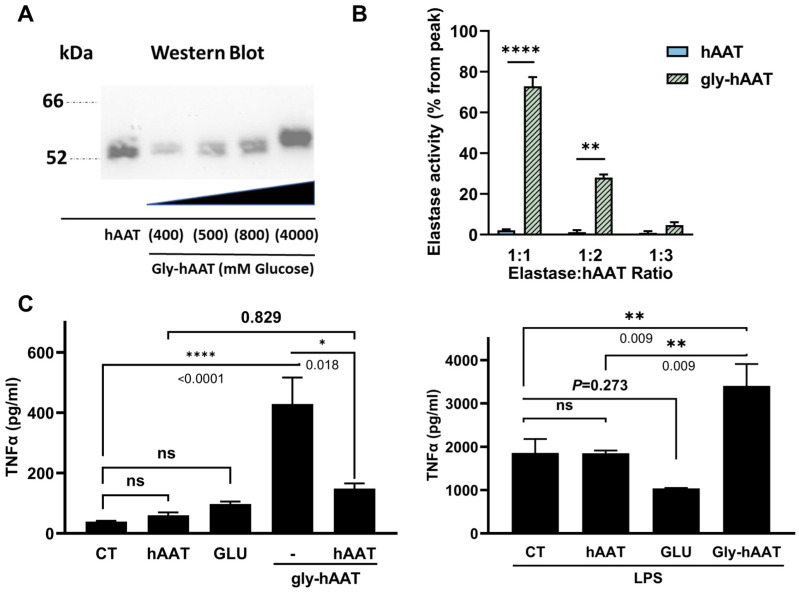
Glycation of hAAT and in vitro analysis of gly-hAAT activities. Clinical-grade hAAT was incubated with the indicated concentrations of glucose for 7 days, followed by extensive dialysis and centrifugal size filtration to remove residual soluble glucose. (**A**) Western blot analysis. A representative blot from 2 independent experiments is shown. Uncropped blots and Coomassie Blue staining are provided in Supplementary Figure S1. (**B**) Elastase inhibitory activity. Concentration gradient using hAAT that is either non-glycated (hAAT) or glycated (gly-hAAT). Data are presented as mean ± SEM from 2 independent experiments, with 4 technical replicates per condition. (**C**) Macrophage stimulation assay. RAW 264.7 cells (25,000 per well in triplicate) were cultured in the absence or presence of LPS (1 ng/mL) for 6 h. Supernatant cytokine levels. Each condition was assayed in technical triplicate. Each group included 8 biological replicates, and the data were generated from 2 independent experiments. Mean ± SEM; ns, non-significant. * *p* < 0.05, ** *p* < 0.01, **** *p* < 0.0001.

A glycated form of hAAT was generated. As shown (Figure 2A), upon prolonged exposure to glucose, hAAT increased in molecular weight in a glucose-concentration–dependent manner. Gly-hAAT generated with 800 mM glucose is used for further investigation; as shown (Figure 2B), naïve hAAT neutralized elastase at a 1:1 molar ratio, while gly-hAAT required a three-fold greater concentrations (72.86 ± 11.00%, 28.06 ± 2.15%, and 4.74 ± 3.06% elastase activity, ×1, ×2, and ×3 gly-hAAT, respectively).

The inflammatory response of the RAW 264.7 macrophage cell line was tested in the presence of gly-hAAT (Figure 2C). Glucose alone was introduced to cells to represent the effect of possible carryover from the glycation protocol. As shown (*left panel*), cells released baseline TNFα levels that were comparable between control, hAAT, and glucose treatments, and cells exposed to gly-hAAT released significantly higher concentrations of TNFα (38.91 ± 7.30 pg/mL and 429.30 ± 247.6 pg/mL, control and gly-hAAT, respectively, *p* < 0.0001). Supplementing gly-hAAT with naïve hAAT significantly reduced TNFα release (147.70 ± 33.86 pg/mL, *p* = 0.018). Under LPS stimulation (*right panel*), TNFα levels increased both in the presence of control medium and hAAT. Glucose reduced TNFα release under LPS stimulation, albeit without reaching statistical significance. TNFα release was further elevated by gly-hAAT (3405.0 ± 1010.0 pg/mL and 856.0 ± 46.4 pg/mL, gly-hAAT/LPS and LPS, *p* = 0.009).

### 3.4. Gly-hAAT Fails to Promote Epithelial Gap Closure In Vitro

**Figure 3:**
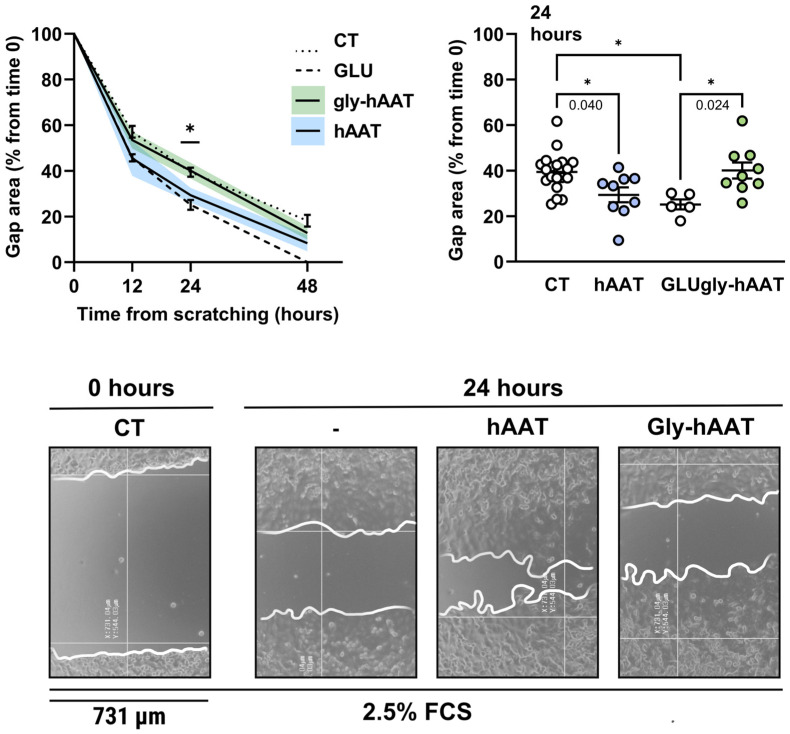
In vitro analysis of the effect of gly-hAAT on wound repair in the epithelial gap closure assay. A549 cells (50,000 cells/well) were disrupted at time 0 and treated with CT (no added treatment; *n* = 19), GLU (80 mg/dL glucose; *n* = 5), hAAT (*n* = 9), or gly-hAAT (hAAT glycated at 800 mM glucose; *n* = 9). (**Bottom**)—representative images with gap borders outlined. The scale bar is set at 731 µm. Data are presented as gap area (% from time 0), mean ± SEM, with deviation marked by solid fill. * *p* < 0.05.

Following assessment of the inflammatory activity of gly-hAAT on macrophages, its impact on epithelial gap closure was investigated (Figure 3). According to the A549 epithelial cell scratch assay, under control conditions, the gap area was reduced to 39.41 ± 8.50% within 24 h, and in the presence of hAAT, it was significantly smaller (29.32 ± 9.80%, *p* = 0.04). Glucose treatment (80 mg/dL) resulted in a residual gap area of 25.14 ± 4.96% at 24 h (*p* = 0.015 compared to control). In contrast, gly-hAAT–treated cells depicted gap closure comparable to control (40.04 ± 10.52% compared to hAAT-treated cells at 24 h, *p* = 0.07).

### 3.5. Gly-hAAT Interferes with Wound Closure in Normoglycemic Mice

**Figure 4:**
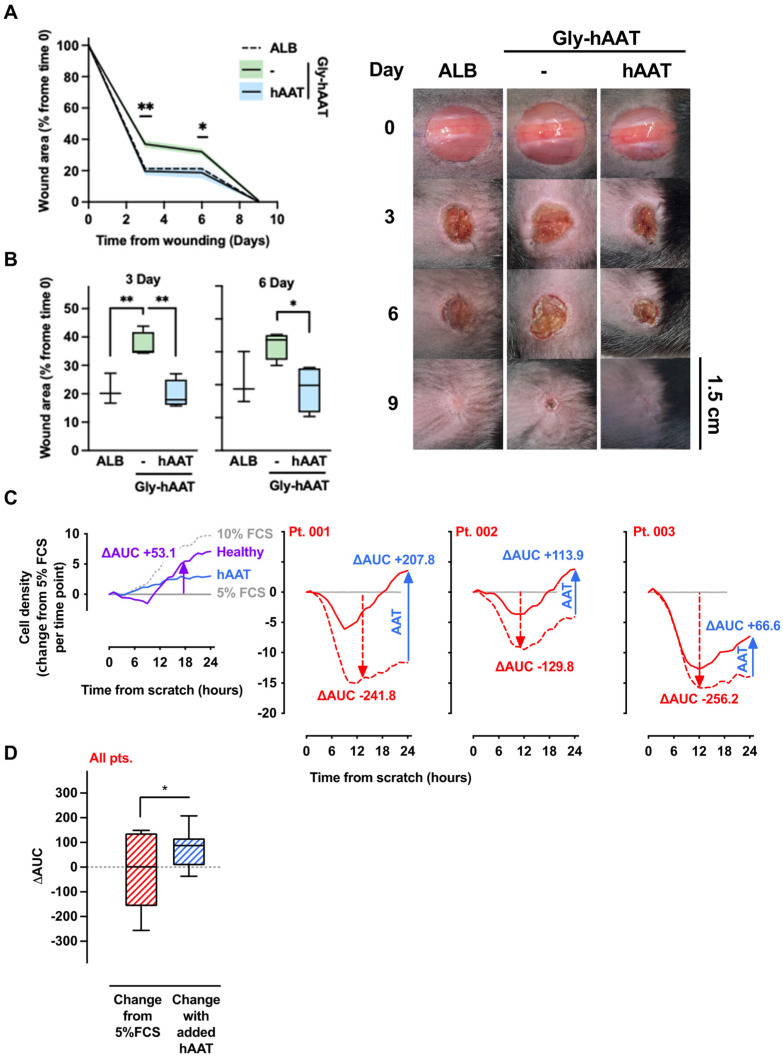
In vivo analysis of the effect of gly-hAAT on wound closure. Normoglycemic mice underwent dorsal excisional wounding and were treated topically with either ALB (albumin), gly-hAAT, or gly-hAAT with added native hAAT (*n* = 5 mice per group). (**A**) Follow-up of wound areas. Images were acquired on days 0, 3, 6, and 9. Data are presented as wound area (% from time 0), mean ± SEM (deviation marked by solid fill). Right, representative images. The scale bar is set at 1.5 cm. (**B**) Wound closure on days 3 and 6. Mean ± SEM, * *p* < 0.05, ** *p* < 0.01. (**C**,**D**) Epithelial gap closure in the presence of sera from patients with poorly controlled diabetes. A549 cells (50,000 cells/well) were disrupted at time 0 and treated with the indicated agents at 5% FCS (set as reference value per time point). Each condition was assayed in 4 technical replicates. Patient sera served as biological replicates (*n* = 10 total), and representative patient sera (*n* = 3) are shown in panel (**C**). (**C**) Epithelial gap closure in the presence of serum from a representative healthy individual (*purple*) or clinical-grade hAAT (*blue*); delta area under the curve (ΔAUC) between 5% FCS gap area and healthy serum per time point (*purple*). Representative patient sera (*n* = 3) are represented by a red dashed line and respective red ΔAUC. *Solid red line*, patient serum supplemented with clinical-grade hAAT; blue ΔAUC indicates the change in impact of patient serum by supplementation with hAAT. (**D**) Grouped data of all 10 patients’ sera. Box plots show ΔAUC relative to 5% FCS and ΔAUC change following the addition of hAAT, with whiskers from min to max. * *p* < 0.05.

Extending on the results obtained with gly-hAAT in vitro, excisional wounds were performed in normoglycemic mice. As shown (Figure 4A), while albumin-treated wounds exhibited 21.35 ± 5.38% area from initial size on day 3, gly-hAAT–treated wounds left an area of 37.00 ± 4.58% from initial wounding (*p* < 0.001). A day-by-day depiction of the differences between groups is presented in Figure 4B. The apparent negative impact of gly-hAAT on wound closure rates was challenged by supplementation with naïve hAAT; accordingly, wound closure was significantly accelerated, resulting in a profile comparable to albumin-treated wounds.

### 3.6. Sera from Patients with Poorly Controlled Blood Glucose Interfere with Epithelial Gap Closure: Attenuation by Clinical-Grade hAAT

Serum was obtained from prospectively recruited patients with poorly controlled diabetes (*n* = 10) and then directly introduced into the epithelial gap-closure assay. Within this cohort, average blood glucose levels were 212.5 ± 98.14 mg%, and HbA1c was 8.0 ± 0.63% (64.0 ± 12.2 mmol/mol; mean ± SD). In addition, clinical-grade hAAT and serum from healthy individuals (*n* = 3) were used for reference; all assays were performed in 5% FCS since a decline in gap closure capacity was anticipated (gap area 5% FCS per time point represented by a gray horizontal line, Figure 4C). As shown, conditions that included healthy serum (*purple*) or hAAT treatment (*blue*) depicted an overall improved cell density compared to 5% FCS alone. In contrast, serum samples from patients with poorly controlled blood glucose (*dashed red line*) appeared to reduce cell density, with each individual, expectedly, being at a unique amplitude (three representative outcomes). The area under the curve (AUC) is calculated for the difference between cell densities at 5% FCS and cell densities under patient serum (*red arrow*), as well as patient serum in the absence or presence of hAAT supplementation (*blue arrow*). As a group (Figure 4D), samples from 10 patients with poorly controlled blood glucose caused a 20.81 ± 47.18 decline in cell density, and adding hAAT increased cell density by 77.5 ± 22.64 (*p* = 0.039).

## 4. Discussion

The effect of a hyperglycemic environment on the properties of hAAT in the context of wound healing was investigated. Experimental findings in the present study show that topical hAAT and transgenic hAAT both enhance the rate of excisional skin wound closure in normoglycemic mice. In hyperglycemic mice, on day 3 from wounding, hAAT-treated wounds showed a higher ratio between tissue IL-1Ra and IL-1β transcript levels. At that time point, hAAT-treated wounds exhibited earlier formation of mature blood vessels compared to controls. Gly-hAAT showed an increased molecular weight, lost its ability to inhibit elastase, and failed to accelerate wound repair in vivo. It stimulated macrophage cultures, even without the presence of LPS, while glucose alone did not elicit such a response. Adding naïve clinical-grade hAAT to gly-hAAT or to human diabetic serum samples partially restored wound healing/epithelial gap closure rates.

The premise of the study is that hAAT acts as a binding protein whose properties are compromised by prolonged exposure to excessive glucose levels. The study further suggests that hAAT glycation takes part in the pathogenesis of difficult-to-heal wounds in patients with diabetes. By this, it is suggested that individuals with poorly controlled glucose levels, at least in the context of tissue injury, may benefit from either topical clinical-grade hAAT or standard hAAT infusion therapy.

That hAAT improves acute wound repair is consistent with the literature. Accordingly, compared to control normoglycemic WT mice, the rate of excisional skin wound closure was enhanced in normoglycemic WT mice treated with topical hAAT and in normoglycemic hAAT^+/+^ mice. To control topical hAAT, human serum albumin was used, as it is similar in molecular weight and, like hAAT, is prone to glycation. It is unclear how hAAT-rich settings speed resolution processes, yet, in their presence, inflammatory signals attract resolution processes sooner [10]. Here, on day 3, wound tissue samples exhibited the predominant transcript levels of IL-1Ra over IL-1β, while, nonetheless, mature blood vessel formation was already observed. These early gene-expression findings should, therefore, be interpreted as limited early measurements that are overall consistent with resolution-associated trends and not as definitive evidence of sustained pro-resolution polarization.

Since protein glycation requires several days and occurs mostly in liver cells, it is believed that newly introduced naïve hAAT remains generally unglycated in the immediate period following treatment [19]. Additionally, hAAT^+/+^ mice produce hAAT in the lung [28].

The observation that gly-hAAT fails to accelerate wound repair fits well with its requirement for intact surface interactions [17]. To explore whether, like other AGEs, glycated hAAT aggravates diabetic wounds, gly-hAAT was generated by exposing clinical-grade hAAT to high levels of glucose over time. Accordingly, its molecular weight increased, it lost its ability to inhibit elastase, and it stimulated macrophage cultures in the absence (and presence) of LPS. Future studies should examine the affinities of binding partners to gly-hAAT. At the functional level, gly-hAAT interfered with wound closure in vivo. Interestingly, gly-hAAT was less of an interruption to cultured epithelial cells, suggesting that in vivo outcomes most probably involve its effect on, at least, macrophages. In both circumstances, however, mixed with naïve clinical-grade hAAT, the negative impact of gly-hAAT was markedly attenuated. In that regard, we asked whether it is possible to rescue the compromised capacity for wound repair contained in serum samples from individuals with poorly controlled blood glucose levels. In a small-scale exploratory clinical arm, clinical-grade hAAT was combined with serum samples from patients with poorly controlled blood glucose levels and then used in an epithelial gap closure experiment. Indeed, patient sera interfered with re-epithelialization, and added hAAT displayed enhanced closure rates, suggesting a reduction in some of the re-epithelialization–limiting factors contained in chronic hyperglycemic serum. Knowingly, this is a qualitative observation and requires a large cohort to explore the phenomenon.

Irrespective of glucose homeostasis, a profound alteration in the functionality of hAAT has been reported in difficult-to-heal wounds. A comparative proteome analysis of wound exudates suggests that hAAT is abundant in acute wounds and scarce in chronic wounds. hAAT exhibits extensive degradation in chronic wounds [24] and decreased activity in venous ulcers [25]. Moreover, we previously demonstrated that oxidative modification of hAAT interferes with its elastase-inhibitory activity and that oxidized hAAT impairs epithelial gap closure and shifts macrophages toward a pro-inflammatory phenotype, thereby compromising tissue repair capacity [34]. Consistently, fluids from chronic wounds exhibit 10–40–fold greater elastase activity than acute wounds [24]. These collective molecular changes in hAAT likely reflect the impact of the local wound microenvironment on its functionality.

Considering the prospect of hAAT as a cell survival and tissue recovery agent, and that pancreatic islet β-cells are targets of autoimmunity in type 1 diabetes, hAAT has been widely tested in both preclinical [35,36] and clinical studies [37,38,39], with beneficial outcomes reported in early-stage disease. Here, animals that received STZ were confirmed to have reached comparable hyperglycemic values across strains at the time of wounding and throughout follow-up. It should be noted, however, that the model was not designed to represent type 1 diabetes but rather to induce hyperglycemic conditions. Accordingly, follow-up duration was constrained by hyperglycemia-related morbidity.

WT mice were studied alongside the transgenic model because a topical approach is more clinically relevant and because hAAT^+/+^ mice are exposed to constant, low levels of ectopic lung-derived circulating hAAT. As such, they do not mimic an infused individual and may somewhat differ from their background WT strain. For example, blood pressure has been shown to be lower in hAAT^+/+^ mice [40,41]. The possibility that mouse anti-hAAT antibodies are induced in WT mice exposed to topical hAAT is unlikely within the time-frame of the present study, as even in high-dose systemic administration, the first signs of anti-hAAT antibodies occur after 18 days [42].

Re-vascularization was examined in the present study at a qualitative level. Histology showed that hyperglycemic mice treated with topical hAAT developed mature blood vessels earlier than controls. This finding is consistent with other reports outside the context of hyperglycemia; Bellacen et al. demonstrated accelerated blood vessel maturation in pancreatic islet allografts [8], and Schuster et al. observed accelerated re-vascularization in a skin flap model [43]. Potential targets for the mechanism of action include endothelial cells, which self-organize and form tubes in culture more rapidly under hAAT-rich conditions, and VEGF, which is expressed earlier in the presence of hAAT [8]. Indeed, hAAT mitigates hypoxia-reperfusion injury and inhibits endothelial cell apoptosis [44], as well as facilitates mature blood vessel formation in normoglycemic settings [45]; importantly, in the case of diabetic retinopathy, enhanced vessel maturation represents a *desired* outcome [46,47]. Interestingly, blockade of VEGF causes emphysema-like features in mice, regardless of intact AAT [48], and in a flap re-vascularization model, the effect of hAAT is only partially impeded by anti-VEGF treatment [43], suggesting that hAAT may promote supportive surroundings for vessel progression rather than directly driving angiogenesis. AGEs increase VEGF expression and are likely present on day 3 wound examination, ten days after STZ was introduced. In this regard, future examination of the impact of hAAT treatment in the flap model under hyperglycemic conditions would hold immense implications for surgical procedures in patients with hyperglycemia.

hAAT increases IL-1Ra levels in different conditions [10,11,12,49], but its ability to do so during hyperglycemia is yet unknown. Here, as all hyperglycemic mice displayed elevated IL-1β transcript levels, IL-1Ra transcript levels set apart hAAT-treated wounds from albumin-treated wounds. In line with the examination of the IL-1 pathway, the expression of genes relating to macrophage polarity was explored. Upon introduction to steady-state RAW 264.7 cells, gly-hAAT elicited an inflammatory response, and in LPS-stimulated conditions, unlike native hAAT, gly-hAAT further aggravated the inflammatory response, agreeing with the general concept of inflammatory AGEs [19] and glycated albumin [26,27]. The TNFα pathway was explored as one that consistently responds to hAAT treatment; specifically, while native hAAT interferes with the TNFα pathway, its glycated form induced TNFα production, even without added LPS. Being that hAAT was shown to bind to and block TNFα receptors, the possibility arises that gly-hAAT fails to bind to TNFα receptors [17,50]. Additionally, lack of protease inhibition by gly-hAAT may contribute to excess release of protease-dependent membrane-bound TNFα [42]. Glucose alone did not elicit a pro-inflammatory response, drawing a clear distinction from the glycation product used in gly-hAAT experiments.

The predominance of M1 macrophages in wound samples under hyperglycemic conditions and the failure of hAAT to promote M2-like macrophages in day 3 wounds stand in contrast to the effects of hAAT in normoglycemic settings [35]. This observation may be attributed to the time point of sampling, when the shift towards M2-like cells is at its very beginning, even in normoglycemic wounds [51]. It is therefore possible that with hAAT treatment, the desired M1/M2 shift in hyperglycemic conditions occurs at a later time point. Further experiments are encouraged for assessing macrophage polarization at wound sites under hyperglycemic conditions and hAAT treatment, as well as using more prolonged experiments with less severe models of hyperglycemia.

While the in vivo arm of the study is by no means a replica of human wounds, it allows for in-depth examination of relevant biological changes. Similarly, the in vitro settings are not intended to represent anything beyond epithelial gap closure under defined conditions and do not aim to recapitulate full human skin wound biology. Together, these models provide a foundation for more complex follow-up studies designed to build on these observations.

It should be strongly emphasized, however, that the model does not represent either type 1 or type 2 diabetes, but rather prolonged hyperglycemic conditions. Glycation of hAAT and poor wound repair are the primary focus of the present study, rather than any particular human condition. Knowingly, the STZ model represents insulin deficiency secondary to loss of pancreatic β-cells, while the present study uses this model to assess straightforward hyperglycemia, a condition more commonly associated with insulin resistance and type 2 diabetes. The choice to use of the STZ model took into consideration the high variability of glycemic profiles of animals in the myriad of models for type 2 diabetes, which, together with the high variability of any wound healing model or, for that matter, human wounds per se, would require a sweeping number of animals. Thus, the hyperglycemic animal model was prioritized over insulin-resistance models. For this reason, in the present study, experimental designs and models are never referred to as anything other than ‘hyperglycemic conditions’. Translatability of the findings to human conditions, particularly in the context of type 2 diabetes, requires disease-directed experiments.

Structural and binding analyses of glycated hAAT were not explored in this study. Future studies should investigate changes in the binding affinities of interaction partners to glycated AAT, as well as its structural profile. Together, these approaches may shed much-needed light on site-dependent functionalities of AAT beyond the reactive center loop.

The exploratory human findings presented herein are to be granted a very modest interpretive weight. At this stage, it is only concluded that diabetic serum interferes with epithelial gap closure and that added clinical-grade hAAT partially mitigates this effect in vitro. There is a need for elaborate experiments to further the conclusions that may be drawn from this initial observation. Accordingly, the potential for clinical translation currently relies primarily on the established safety of hAAT infusions in humans, rather than on robust experimental evidence.

## 5. Conclusions

In conclusion, the present study supports a role for clinical-grade hAAT in promoting wound repair under hyperglycemic conditions across complementary in vivo and in vitro models, while suggesting that glycation may impair its protective functions and promote pro-inflammatory activity. These findings provide mechanistic support for the concept that glucose-driven modification of hAAT may contribute to impaired wound healing in clinical diabetes, and support continued investigation of hAAT as a potential therapeutic strategy for impaired wound healing in hyperglycemia.

## Data Availability

The raw data supporting the conclusions of this article will be made available by the authors on request. This study is reported in accordance with the ARRIVE guidelines. A completed ARRIVE checklist has been uploaded.

## Supplementary Materials

The following supporting information can be downloaded at https://www.mdpi.com/article/10.3390/biology15080606/s1, Figure S1. Uncropped Western blot images corresponding to Figure 2A.
