# Supplementary material for: Glycated and Non-Glycated Human Alpha-1 Antitrypsin in Hyperglycemic Wound Healing: In Vivo and In Vitro Models
Source: Biology (Basel). 2026 Apr 11;15(8):606. doi: 10.3390/biology15080606 (PMC13113610; doi:10.3390/biology15080606)
Supplement: Supplementary file 1 [file biology-15-00606-s001.zip › biology-4166477-supplementary.pdf]

Supplementary Figure S1

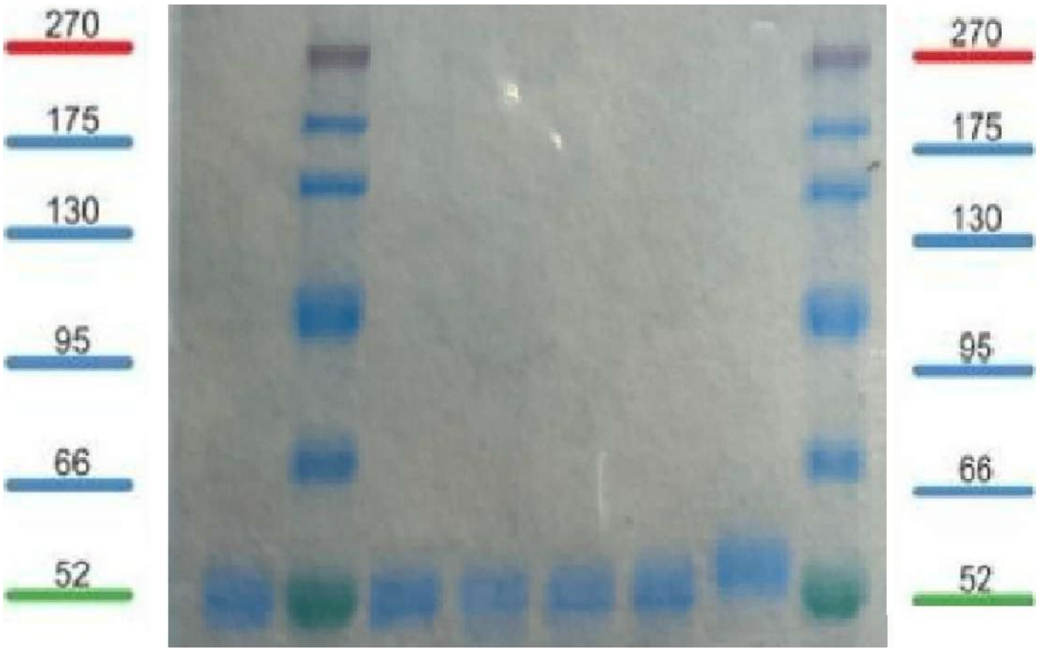

Glassia Gly-a Gly-400 Gly-500 Gly-800 Gly-4000

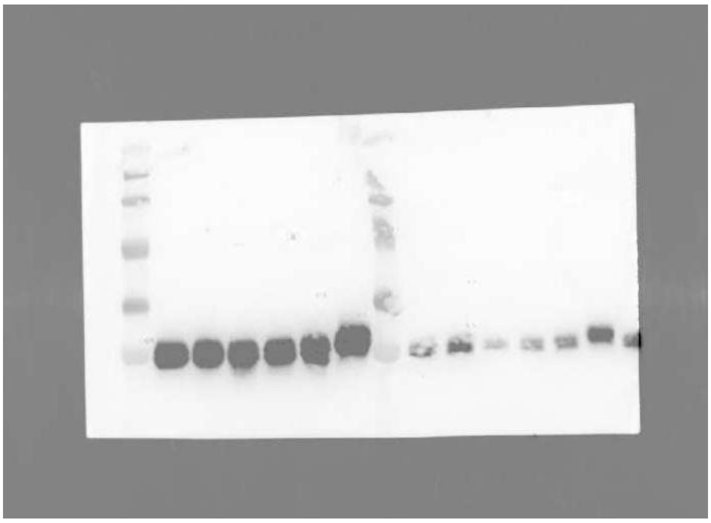

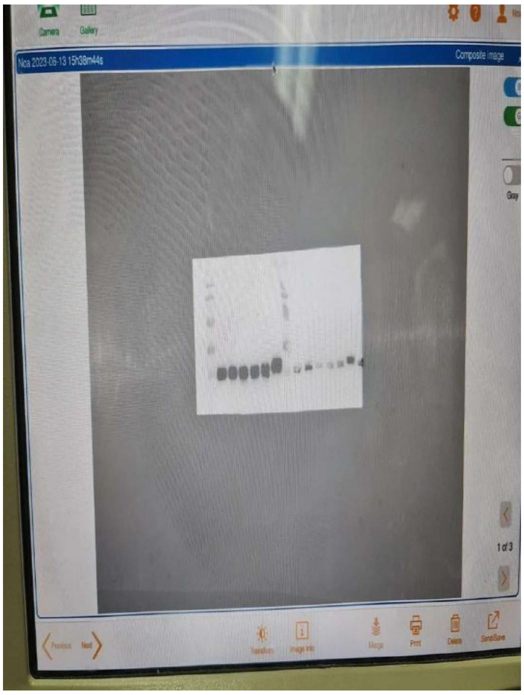

Supplementary Figure S1. Uncropped Western blot images corresponding to Figure 2A. Full, uncropped membranes of the Western blot analysis presented in Figure 2A showing hAAT following incubation with increasing glucose concentrations.
